# The Structure Design and Photoelectric Properties of Wideband High Absorption Ge/GaAs/P3HT:PCBM Solar Cells

**DOI:** 10.3390/mi13030349

**Published:** 2022-02-23

**Authors:** Xintao Zeng, Ning Su, Pinghui Wu

**Affiliations:** Fujian Provincial Key Laboratory for Advanced Micro-Nano Photonics Technology and Devices & Key Laboratory of Information Functional Material for Fujian Higher Education, Quanzhou Normal University, Quanzhou 362000, China; liubin@lyu.edu.cn (X.Z.); qsz20211397@student.fjnu.edu.cn (N.S.)

**Keywords:** hybrid solar cell, Ge/GaAs/P3HT:PCBM, localized surface plasmon, photon absorption, FDTD

## Abstract

Using the finite-difference time-domain (FDTD) method, we designed an ultra-thin Ge/GaAs/P3HT:PCBM hybrid solar cell (HSC), which showed good effects of ultra-wideband (300 nm–1200 nm), high absorption, and a short-circuit current density of 44.7 mA/cm^2^. By changing the thickness of the active layer P3HT:PCBM, we analyzed the capture of electron-hole pairs. We also studied the effect of Al_2_O_3_ on the absorption performance of the cell. Through adding metal Al nanoparticles (Al-NPs) and then analyzing the figures of absorption and electric field intensity, we found that surface plasma is the main cause of solar cell absorption enhancement, and we explain the mechanism. The results show that the broadband absorption of the solar cell is high, and it plays a great role in capturing sunlight, which will be of great significance in the field of solar cell research.

## 1. Introduction

With the increasing shortage of coal, oil, and natural gas and the strong support of new energy, solar energy, as a clean and pollution-free renewable energy with rich resources, has become a hot research topic among scholars [1,2,3,4]. Although silicon is the most widely used commercial material for solar cells [5,6], GaAs has its own advantages. GaAs bandgap is 1.42 eV, and Si bandgap is 1.12 eV. GaAs has a high electron mobility, up to 8000 cm^2^/V·s; the electron mobility of silicon is 1350 cm^2^/V·s. Thus, GaAs has a wider bandgap and higher absorption coefficient than Si. A photoactive layer of several microns is enough to use the transmitted light, because of the direct band gap in GaAs as compared to Si or other semiconductor materials. Moreover, GaAs is widely studied in solar cells because of its high electron saturation rate and electron mobility [7]. Pham et al. designed a positive ZnO electronic-selective contact single-junction GaAs cell with a current density of 30.48 mA/cm^2^ [8]. In order to reduce the substrate cost, GaAs solar cells usually have Ge as the substrate material [9,10,11]. Ge is commonly used in the wafer substrates of high-efficiency multi-junction photovoltaic cells for space applications [12,13]. In addition, Ge and GaAs have similar lattice constants; their lattice constants are 5.646 and 5.653, thus avoiding excessive lattice imperfection from being introduced on the back surface [14]. There are Mars rovers and several satellites that use three-junction GaAs on Ge cells. Thus, Ge substrate is utilized to manufacture GaAs solar cells [15]. A good passivation layer is essential for solar cells. Al_2_O_3_, with excellent field-effect passivation properties and good chemical passivation properties, has been widely studied as a possible gate oxide of GaAs [16], which plays a certain role in improving the efficiency of solar cells. Polymer material P3HT:PCBM has improved carrier mobility, so we combined polymer materials and semiconductor materials to form a hybrid solar cell.

In addition to adding passivation layers, there are many ways to improve the efficiency of solar cells, such as nanomaterials [17,18], anti-reflection films [19,20], surface texture [21,22], etc. Among them, metal nanostructures have been widely studied in the field of solar cells due to their unique properties of enhancing electric fields and improving light absorption [23,24,25]. They use the local electromagnetic field near the metal surface to amplify and produce local surface plasmons (LSPs) at the interface between metal nanoparticles and semiconductor materials [26,27,28]. The electromagnetic wave propagated by LSPs along with the interface of the metal back contact and semiconductor absorption layer can effectively capture and conduct incident light in the semiconductor layer [29]. Ding Dong et al. studied the light absorption of microcrystalline silicon thin film solar cells enhanced by Al nanoparticles, and obtained different ratios of radii and periods, resulting in different surface plasmon resonance effects [30]. Different sizes, shapes, and arrangements of metal nanoparticles affect the excitation of these excitons and thus the absorption of solar cells [31,32,33]. Gurjit Singh et al. discussed the absorption enhancement effect of copper particles with sizes of 40–200 nm on GaAs solar cells. They also discussed the effect of Al nanoparticles with different sizes on the front and back surfaces to enhance the surface plasmon absorption of GaAs solar cells based on surface plasmon absorption [34,35]. For this reason, we performed a detailed study and analysis of the metal nanoparticles of the HSC. 

In this paper, we use FDTD Solutions to simulate the Ge-based GaAs-P3HT:PCBM HSC based on Al-NPs. By introducing the spherical Al-NPs model based on FDTD, it is found that LSP is the main reason for the enhancement of solar cell light absorption, and its mechanism is analyzed. It is shown that this three-dimensional Al-NPs optical model is very useful for understanding the light absorption behavior of Al-NPs in solar cells varying with wavelength and shape parameters. We also studied the absorption characteristics of the organic active layer of P3HT:PCBM deposited on the GaAs surface to analyze the specific mechanism.

## 2. Structure Design and Numerical Model

**Figure 1:**
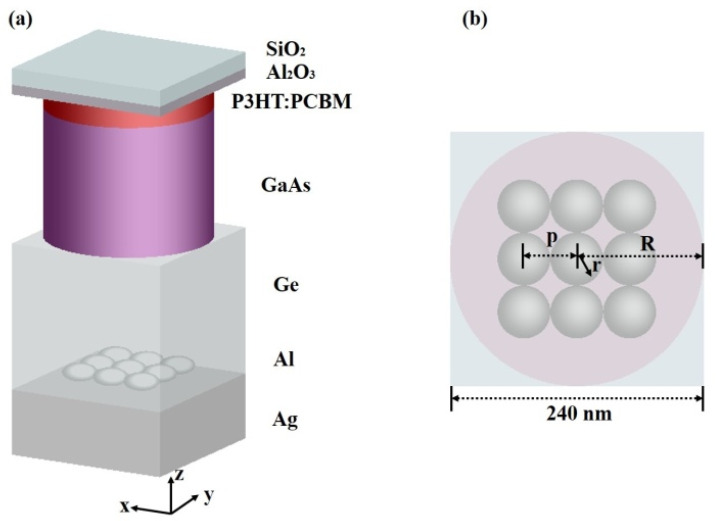
(**a**) HSC 3D diagram. (**b**) Plan of HSC in the x-y plane.

The structure of the cell is shown in Figure 1. Ag is the backplate, and Ge is the substrate to produce the plasma plasmon metal aluminum nanoparticles of the GaAs-P3HT:PCBM hybrid solar cell. The Ag layer thickness is 200 nm, and the Ge layer thickness is 450 nm. The thickness of the GaAs cylinder is 400 nm, with a radius of 120 nm. The thickness of P3HT:PCBM of the cylinder is 60 nm to capture photons, and the thickness of Al_2_O_3_ as the gate oxidant is 30 nm to protect it. Since the FDTD calculation can only be carried out in a limited area, periodic boundary conditions are set on edges of x and y to avoid the generation of reflected waves, and a high matching layer (PML) [36] is used at the top and bottom of the *z*-axis. The plane-wave excitation source is placed directly above the model to simulate the solar light source, and a reflective power monitor and transmission power monitor are placed to calculate the reflectivity R and transmittance T, respectively. According to A = 1-R-T, the absorption rate of the battery can be obtained [37]. The current density value can be calculated by setting the analytical photon production rate.

FDTD Solutions was used to simulate the solar cell. The FDTD method, proposed by Kane Yee [38,39], is a differential differentiation of Maxwell equations in time and space. The electric field and magnetic field are calculated alternately in the space domain and updated in the time domain to simulate the change of electromagnetic field, to achieve the purpose of numerical calculation [40,41]. PML is used for special processing in the limited space so that the internal field does not produce distortion. The carrier generation rate can be calculated with FDTD, and its expression is [42,43]

(1) $$G\left(\vec{r}\right)=\frac{P_{abs}\left(\vec{r},\omega \right)}{\hbar \omega }=\frac{-0.5{\left|E\left(\vec{r},\omega \right)\right|}^{2}Im\left[\varepsilon \left(\vec{r},\omega \right)\right]}{\hbar }\;\;\;\;\;$$

*P_abs_* is the absorption space power density, and its expression is [42]

(2) $$\;\;\;\;\;\;\;\;\;\;\;\;\;\;\;\;\;P_{abs}\left(\lambda \right)=-0.5\omega |E\left(\lambda \right){|}^{2}Im\left[\varepsilon \left(\lambda \right)\right]\;\;\;\;\;\;\;\;\;\;\;\;\;\;$$

where *ω* is the angular frequency, *E* is the electric field intensity, *ε* is the permittivity, and *ℏω* is the energy of a photon. The FDTD software then calculates the short-circuit current density directly:

(3) $$\;\;\;\;\;\;\;\;\;\;\;\;J_{sc}=\frac{e}{\hbar c}\int_{300\;\mathrm{nm}}^{1200\;\mathrm{nm}}QE\left(\lambda \right)\lambda I_{AM1.5}\left(\lambda \right)d\lambda \;\;\;\;\;\;\;\;\;\;\;\;\;\;\;$$

where *ℏ* is reduced Plank’s constant, *c* is the speed of light in the free space, *QE* is the Quantum Efficiency of Solar Cell, and *I_AM_*_1.5_ is the AM1.5 solar spectrum [44].

## 3. Simulation Results and Discussion

**Figure 2:**
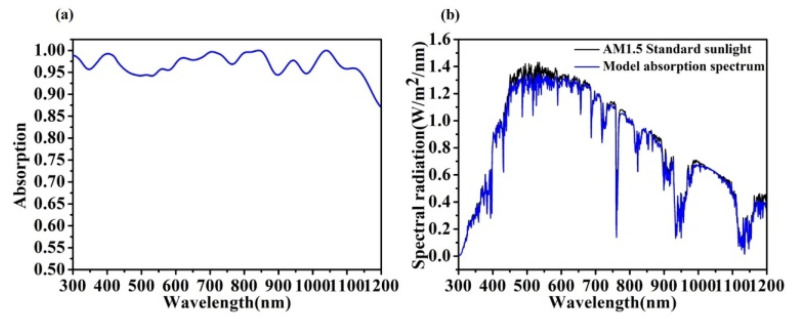
(**a**) HSC absorption curve; (**b**) AM1.5 standard sunlight, and the HSC absorption spectra.

AM1.5 is the average illumination of sunlight incident on the earth’s surface, and its total solar radiation is 100 mW/cm^−2^ [45]. The light absorption rate of the solar cell designed after optimization is shown in Figure 2a. Within 300 nm–1200 nm, the light absorption rate of the cell is above 90%, and most of the light absorption is above 95%, which can be approximated to the high absorption of broadband. Figure 2b shows the absorption spectrum of the cell under the standard spectrum AM1.5, and the absorption spectrum of the cell is very close to the curve of AM1.5. Figure 2 shows that the solar cell has a good absorption of sunlight and can capture photon energy well, thus improving the conversion efficiency of the cell from the source [46,47].

**Figure 3:**
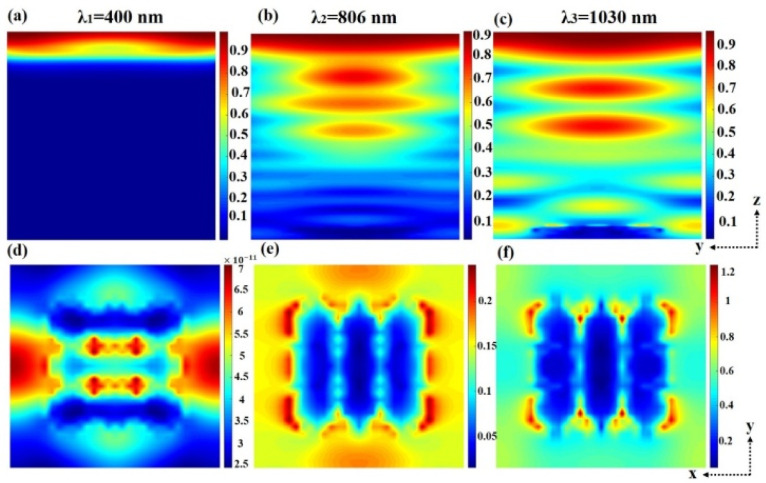
The electric field distribution in the y-z plane at three wavelengths: (**a**) 400 nm, (**b**) 806 nm, (**c**) 1030 nm, with an absorption intensity greater than 97%. The electric field action of Al-NPs in the x-y plane at three wavelengths: (**d**) 400 nm, (**e**) 806 nm, (**f**) 1030 nm.

Figure 3 shows the electric field intensity at three values of the highest absorption rate: λ1, λ2, and λ3. Figure 3a–c reflects the electric field intensity of the longitudinal section z from 200 nm to 1190 nm, that is, the electric field intensity of the y-z plane. The electric field intensity gradually increases from left to right. When λ1 = 400 nm, the electric field intensity of the structure remains unchanged. In Figure 3b,c, the longer wave, because of its stronger diffraction ability, after the part is absorbed by the material energy; the residual electromagnetic energy had a deeper extrinsic absorption, producing the electric field intensity distribution in the graph. The light spread resulted in the light carrier moving up, making the electric field enhancement red. From Figure 3b, λ2 = 806 nm, and from Figure 3c, λ3 = 1030 nm, showing that the electric field intensity between GaAs and P3HT:PCBM is enhanced. A high electric field will speed up the transport between electrons and holes and avoid electron-hole recombination [48]. We can see that there is obvious diffraction enhancement in the bottom layer, which is the result of surface plasma element interaction. Figure 3d–f reflects the cross-section at z = 200 nm and the electric field intensity on the x-y plane. As can be seen from the figure, Al-NPs interact with the medium to produce the plasma plasmon, which strengthens the electric field vibration [49].

**Figure 4:**
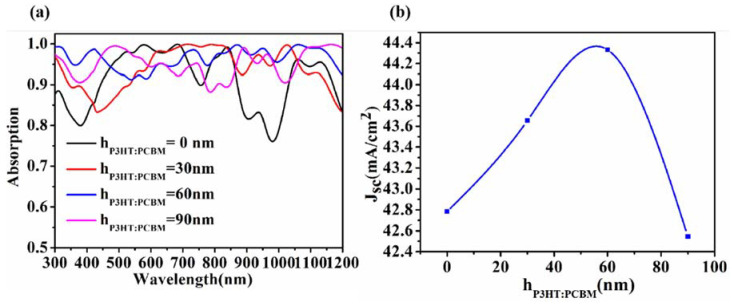
Effect of different P3HT:PCBM thicknesses on light absorption (**a**) and current density (**b**).

Figure 4 shows the influence of the thickness of P3HT:PCBM on the absorption and current density of the solar cell. P3HT:PCBM has chemical stability and high crystallinity carrier mobility and absorption, and it is the most commonly used material applied to organic photovoltaics (OPVs) [50]. Compared with the absence of P3HT:PCBM(h_P3HT:PCBM_ = 0 nm), the addition of P3HT:PCBM enhanced the cell absorption at 300 to 500 nm and 850 to 1050 nm. With the increase of h_P3HT:PCBM_, the photon absorption rate increases gradually. When h_P3HT:PCBM_ = 60 nm, the photon absorption rate is increased. However, when h increases again, the absorption rate of photons decreases between 800 and 900 nm, indicating that the excessively thick P3HT:PCBM hinders the transmission of photons and affects the absorption rate of solar cells. The current density J_SC_ increases first and then decreases with the increase of h_P3HT:PCBM_, indicating that the thickness of P3HT:PCBM has an influence on the ability of electron and hole transport, and excessive thickness will hinder the transport of electrons and holes. Therefore, we chose a P3HT:PCBM thickness h_P3HT:PCBM_ of 60 nm as the optimal parameter to increase the photon absorption effect for the cell.

**Figure 5:**
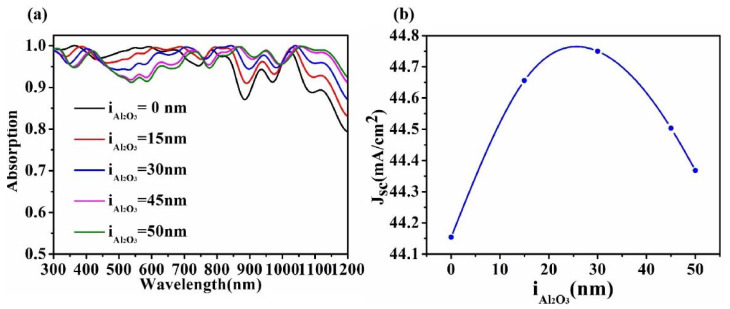
Absorption (**a**) and current density (**b**) of Al_2_O_3_ with different thickness (h_P3HT:PCBM_ = 60 nm).

Figure 5 describes the influence of Al_2_O_3_ on the performance of the cell. As a reflection reducing layer, Al_2_O_3_ is often placed on the surface of GaAs to reduce reflection and increase absorption. Compared with the cells without Al_2_O_3_ (i_Al2O3_ = 0 nm), the cells with the Al_2_O_3_ anti-reflection layer have absorption enhancement in the near-infrared band. It reduces the reflection of photons and allows more of the photon energy to be injected into the cell, thus increasing absorption. With the increase of i_Al2O3_, the absorption of the near-infrared band gradually increases, but at the same time, the absorption of the visible band gradually decreases. The results show that the anti-reflection effect of Al_2_O_3_ gradually moves to the infrared band with the increase of its thickness, and it can increase the absorption of long wavelengths. With the increase of the thickness of Al_2_O_3_, the field passivation effect is significant and the collecting efficiency of the carrier is improved; thus, the short-circuit current is increased. When the thickness of Al_2_O_3_ is too high, the minority carrier life is not enough to support the carrier transmission, and the current density decreases [51]. Therefore, the i_Al2O3_ = 30 nm anti-reflection layer was selected to improve the overall performance of the HSC.

**Figure 6:**
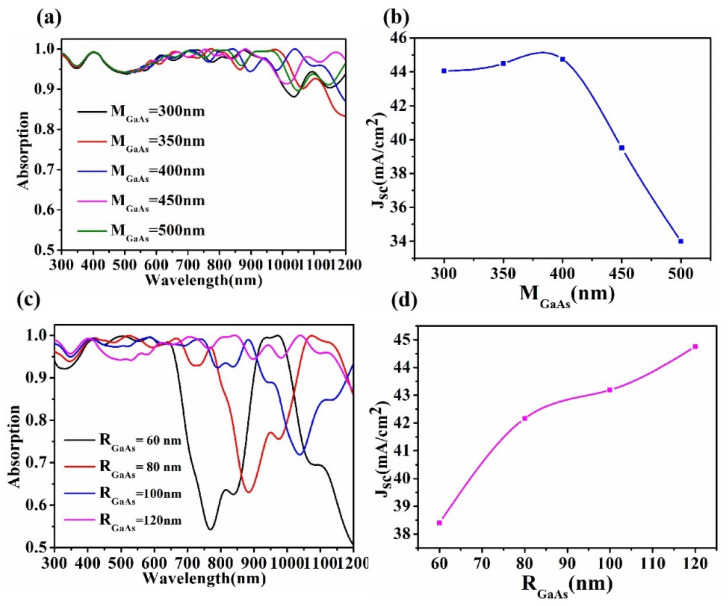
Changes in absorption (**a**) and current density (**b**) with GaAs thickness. Diagram of changes in absorption (**c**) and current density (**d**) caused by cylindrical GaAs radius (h_P3HT:PCBM_ = 60 nm, i_Al2O3_ = 30 nm).

In Figure 6a, in the visible band, the thickness of GaAs has little influence on light absorption. The absorption properties of GaAs in the near-infrared band are different with the change of thickness. In Figure 6b, the current density is the highest when M_GaAs_ = 400 nm. As M_GaAs_ continues to increase, the electron and hole transport distance in GaAs is longer, and the current density decreases. Therefore, 400 nm thick GaAs was selected as the optimal parameter. Figure 6c plots the absorption of the GaAs radius to the solar cell. The larger the radius, the stronger the absorption capacity, especially in the 700–1200 nm band. J_SC_ shows an upward trend with the increase of R_GaAs_, as shown in Figure 6d. The larger radius can make the carrier transport capacity in the semiconductor stronger and avoid the recombination of electron hole pairs in the small region, and thus can improve the short-circuit current density. Therefore, when the radius of GaAs of the cylinder is R_GaAs_ = 120 nm and the thickness is M_GaAs_ = 400 nm, the absorption effect and current density are the best.

**Figure 7:**
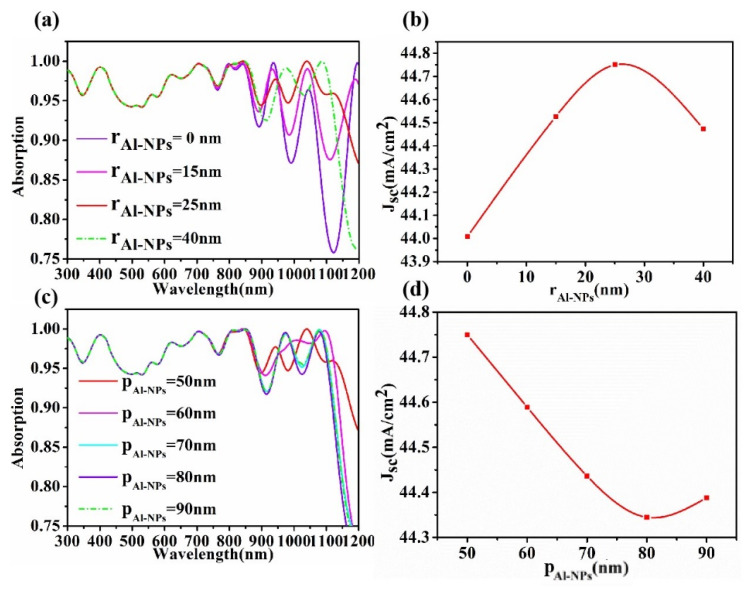
Changes in absorption (**a**) and current density (**b**) due to different sizes of Al-NPs. Influence diagram of solar cell absorption (**c**) and current density (**d**) caused by Al-NPs spherical center spacing (h_P3HT:PCBM_ = 60 nm, i_Al2O3_ = 30 nm, M_GaAs_ = 400 nm, R_GaAs_ = 120 nm).

Because the interface between the metal and the medium will produce plasma plasmons, the action of electromagnetic wave and electromagnetic field will be changed, so we explored the influence of the size and spacing of Al-NPs on the solar cell (see Figure 7). In Figure 7a, with the increase of radius r_Al-NPs_, the light absorption rate of the cell gradually increases in the near-infrared band. The results show that the absorption of Al nanoparticles increases obviously in the long wavelength band. Figure 7b shows that J_SC_ increases first and then decreases with the increase of r_Al-NPs_, and its maximum value is around r_Al-NPs_ = 25 nm. Because the particle size of Al nanoparticles has a great influence on the spectral absorption rate of the cell surface in the near infrared band, the lower the absorption rate is, the fewer the photons that are captured and the fewer the electrons that are transmitted, resulting in a lower current density [52,53]. Figure 7c shows the effect of p_Al-NPs_ differences in the centroid spacing between two Al-NPs. In general, spacing p has little influence on the light absorption rate of the cell, but a great influence on the current density. As can be seen from Figure 7d, when the two spheres are close to each other (p_Al-NPs_ = 50 nm), the effect is the strongest and the current density is the largest.

**Figure 8:**
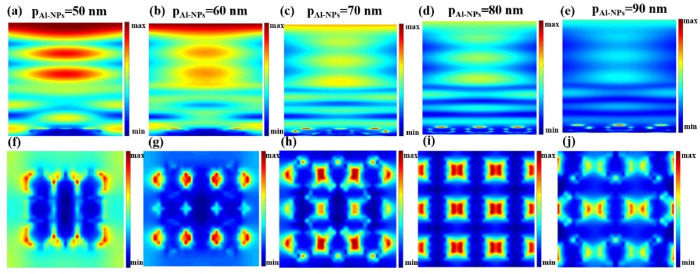
The electric field of the HSC at (**a**) p_Al-NPs_ = 50 nm, (**b**) p_Al-NPs_ = 60 nm, (**c**) p_Al-NPs_ = 70 nm, (**d**) p_Al-NPs_ = 80 nm, and (**e**) p_Al-NPs_ = 90 nm in y-z plane. (**f**–**j**) Electric field distribution of Al-NPs with different center spacing in the x-y plane.

Figure 8 shows the electric field diagram of the spherical spacing between two aluminum particles at λ = 1030 nm. Figure 8a–e is the electric field diagram on the y-z plane of the longitudinal section. When p_Al-NPs_ = 50 nm, the effect between adjacent aluminum spheres intensifies the electric field vibration, and the electric field intensity is the highest, which is the highest consistent with the absorption rate in Figure 7c. Figure 8f–j is the electric field diagram of the cross-section x-y plane at the center of the Al-NPs. It shows the excitation of LSP near the Al as a function of the spacing between the Al-NPs. In Figure 8f, the adjacent Al spheres generate a strong electric field and propagating waves, which spread around to enhance light absorption and thus increase the photocurrent. In Figure 8g–f, the Al spheres are not adjacent and only produce plasmons locally, with a very small acting region. In Figure 8j, due to the distance, the electric field oscillation effect is not strong, resulting in a weak electric field.

## 4. Conclusions

In this paper, the GaAs thickness and size of the solar cell, active layer thickness P3HT:PCBM, metal nanoparticles, and other structural parameters were studied. The mechanism of photon capture by P3HT:PCBM and the influence of surface plasmon on the solar cell were analyzed. Based on the above numerical simulation, we designed an optimal ultra-thin GaAs/P3HT:PCBM hybrid solar cell based on germanium substrate plasma plasmon. The broadband high absorption of this solar cell will allow further breakthroughs in this field. With the deepening of research and the continuous progress of technology, this type of solar cell will make greater progress and be widely used in the field of solar cells.

## Data Availability

Not applicable.
